# Frequency of premolar teeth extractions for orthodontic treatment

**DOI:** 10.6026/973206300161080

**Published:** 2020-12-31

**Authors:** Anisha Mahtani, Ravindra Kumar Jain

**Affiliations:** 1 Saveetha Dental College, Saveetha Institute of Medical and Technical Sciences, Saveetha University, Chennai 77, India

**Keywords:** Angle's malocclusion, Crowding, Orthodontic extractions, Premolar extractions

## Abstract

It is of interest to evaluate the frequency of premolar extractions during orthodontic treatment in patients reporting to the Saveetha dental hospital in Chennai from 2019-2020. We used the records from 987 patients who underwent orthodontic treatment from June 2019 to March 2020 in a dental hospital for this analysis. Digital case records of patients who underwent therapeutic extractions of premolars were isolated. A sample dataset of 340 case records were selected for this study. Data shows that 34.4% of subjects underwent premolar extractions amongst a total of 987 subjects who underwent orthodontic treatment. 89.4% of patients were Angle's Class I malocclusion patients, and the rest were Class II patients. However, no premolar extractions were done in Class III patients. Data also shows that 67.1% of subjects underwent all 4 first premolar extractions and 13.2% underwent only upper first premolar extractions. Thus, a significant association was found between Type of Malocclusion and the Type of premolar extractions with p < 0.05. Moreover, only 34.4% of patients underwent premolar extractions and the majority of them underwent all 4 first premolar extractions.

## Background

Orthodontic treatments commonly involve premolar extractions for providing space and are known as therapeutic extractions. First premolar extractions are commonly preferred to correct crowding and proclination of anterior segment. One of the first orthodontists to indicate permanent tooth extractions to correct malocclusions was Charles Tweed, who found only 20% of his clinical cases without extractions were successful [1]. His ideas were however considerably different from the non-extraction theory supported by Edward Angle. Premolar extractions are currently well accepted in the treatment of cases of malocclusion that include severe crowding, bimaxillary protrusion, convex facial profiles and large cephalometric discrepancies. Tooth crowding and protrusions demand rigorous attention during orthodontic planning and may include the extractions of 1st and 2nd premolars 2. In previous studies done it was reported that in the University of North Carolina from 2000-2011 the overall extraction rates declined from 37.4% to 25%. They also stated an overall decline of four first premolar extraction rates from 16.5% to 12.4 % [2]. With the advent of self-ligation brackets especially Damon philosophy non-extraction treatment plans have become more common among orthodontists especially for cases, which fall under the borderline category in space analysis. Extraction rates were also found to be higher in non-Caucasian participants [3]. Therefore, it is of interest to evaluate the frequency of premolar extractions during orthodontic treatment in patients reporting to the Saveetha dental hospital in Chennai from 2019-2020.

## Methodology

### Study design and Study setting:

This retrospective cross-sectional study was conducted in Saveetha dental college and hospital, Saveetha University, Chennai, to evaluate the frequency of premolar extractions during orthodontic treatment for a period of 10 months from June 2019 to March 2020. The retrospective study was carried out using the digital case records of 987 patients who underwent orthodontic treatment in the hospital. Since it is a retrospective study, carried out using digital case records, no informed consent was required from the patient. Ethical clearance to conduct this study was obtained from the Institutional Review Board of the hospital with the following ethical approval number - SDC/SIHEC/2020/DIASDATA/0619-0320.

### Sampling:

After assessment in the university patient data registry, case records of 340 patients who underwent premolar extractions were included in the study. The exclusion criteria was missing or incomplete data. Only relevant data was included to minimize bias and non-probability sampling method was carried out. Cross verification of data for errors was done with the help of clinical photographs. The study contained regional data generalised to the South Indian population.

### Data Collection:

A single examiner evaluated the digital case records of the patients from June 2019 to March 2020. Case records of patients who underwent premolar extractions for orthodontic treatment were isolated. The examiner evaluated the strap up photographs of the 340 patients from the digital case records to categorise the different types of premolar extractions carried out during treatment. Premolar extractions were categorised into 9 types: All first premolar extractions (All 4), Upper 1st premolar and lower 2nd premolar (U4L5), All upper first premolar extractions (U4), Lower 1st premolar extractions and upper 2nd premolar (L4U5), All 2nd premolar extractions (All 5), All upper 2nd premolars (U5), All lower 1st premolar extractions (L4) and Unilateral and Asymmetric type of premolar extractions. Demographic details like age, gender were also recorded.

### Statistical Analysis:

The collected data was validated, tabulated and analysed with Statistical Package for Social Sciences for Windows, version 20.0 (SPSS Inc., Chicago, IL, USA) and results were obtained. Categorical variables were expressed in frequency and percentage; and continuous variables in mean and standard deviation. Chi-square test was used to test associations between categorical variables. Chi Square tests were carried out using age and gender as independent variables and Types of Premolar extractions as Dependent variables. P value < 0.05 was considered statistically significant.

## Results:

Out of the 987 patients undergoing orthodontic treatment in the hospital, this study found only 340 case records (34.4%) of treatments involving premolar extractions. The study involved 57.9 % of females and 42.1% of males undergoing premolar extractions ranging from age 11 to 45 years. The 16 to 20 year age group had a maximum number of patients; 32.9% undergoing premolar extractions (Figure 1 and Table 1 - see PDF). Regarding the types of premolar extractions; All first premolar extractions (All 4) were found to be the maximum at 67.1% followed by U4 at 13.2%, Asymmetric extractions at 10% and U4L5 at 4% (Table 1 - see PDF). The type of malocclusion in patients undergoing therapeutic premolar extractions were maximum in Angle's Class I Malocclusion at 89.4%, the remaining patients had Class II Malocclusions and no premolar extractions were found to be done in Class III patients (Table 1 - see PDF). While analysing the comparison between the age of patients with Type of malocclusion, a higher prevalence of Class 1 malocclusion in all age groups was seen. On statistical comparison using Chi Square tests, no significant association was obtained between age of patients with Type of malocclusion with p=0.287 (Figure 2 - see PDF). The comparison between the age of patients and Type of premolar extractions showed a higher prevalence of All first premolar extractions (All 4's) in all age groups. Statistical analysis depicted no significant association between age of patients with Type of extractions with p=0.793 (Figure 3 - see PDF). The comparison between the gender of patients and Type of premolar extractions showed a higher prevalence of all types of premolar extractions in females than in males. Statistical analysis depicted no significant association between gender of patients with Type of extractions with p=0.791 (Figure 4 - see PDF). On performing the association of the type of extraction with the type of malocclusion, a drastically higher prevalence of All 4 premolar extractions in Class I malocclusion patients was seen followed by only U4 extractions and asymmetric extractions were also higher in Class I patients than Class II. The study found a significant association between Type of Malocclusion and the Type of premolar extractions on statistical analysis with p < 0.001 (Figure 5 - see PDF).

## Discussion:

This study was carried out to evaluate the frequency of premolar extractions for orthodontic treatment in patients reporting to a dental hospital, which revealed that only 34.4% of patients underwent premolar extractions and the majority of them underwent all 4 first premolar extractions. The decision for extraction in orthodontics is challenging especially for borderline cases. Clinicians would recommend premolar extractions for patients with steep mandibular planes, increasing facial height and even with minor dentoskeletal discrepancies. The philosophy behind this treatment protocol is that the extractions would provide forward and upward movement of molar teeth [4,5]. On gender comparisons, this retrospective study found a higher prevalence of therapeutic premolar extractions in females (57.9%) than in males (42.1%) (Table 1 - see PDF). This statement was supported by studies done by Peck S [6], with higher extraction rates in females (44%) than males (39%) and by Jackson TH [2] with extraction rates in females at 58.3% and males at 41.7%. However, this finding was opposed by another clinical study that found males with a higher rate of extractions (48%) than females (44%). Jackson TH [2] in his study conducted in March 2010 reported that maximum number of patients pursued orthodontic treatment at 14 years of age, but according to this study, maximum number of patients orthodontically treated was in the 16-20 year age group (Table 1, Figure 1 - see PDF). This study found the most frequently extracted teeth to be the first premolars (Table 1 - see PDF) and supported by many clinical studies [2,7-13]. This finding could be due to the location of these teeth in the dental arch nearest to the area of discrepancy, which is most commonly the anterior region. From 2003 to 2007, the rate of type of premolar extractions as reported by Janson G [14] in his study was, first premolar extractions (All 4) at 7.47%, upper first premolars and lower second premolar at 1.3%, all second premolar extraction rates at 1.95% and Asymmetric extractions at 6.82% In 2019 to 2020, the present study revealed the rate of all first premolar extractions to be 67.1%, with upper first premolar and lower second premolar at 4.1%, all second premolar extractions at 2.9% and Asymmetric extractions at 10%. This is mainly due to the majority of the patients reporting to the present hospital set up were diagnosed as Class I malocclusion with crowding and bimaxillary protrusion. This study shows an increase in the rates of types of extractions compared to the study done by Janson G in 2003 to 2007 [14]. This is due to the difference in ethnicity and predominant malocclusions reported in the present study. A gradual increase is seen in the extraction rates with all first premolar extractions always being the most common type. The current study found maximum extractions done in Class I patients (89.4%) with 10.6% of extractions in Class II patients. (Table 1 - see PDF) The percentage of cases with Class II was very less in this study and this could be the reason for the discrepancy. No premolar extractions were found in Class III patients in this study. This statement was supported by MorieraTC [15], who also found maximum extractions done in Class I patients (68.6%). However, the result of this study was contradicted by another study [7] that not only found a higher rate of extractions being done in Class II patients rather than Class I, they also found 9.2% of Class III patients undergoing extractions. Jackson TH [2] also found a higher number of extractions being done in Class II patients (46.3%) than Angle's Class I patients (42.3%). On statistical analysis, non-significant associations were made between the Types of malocclusion with age of patients (Figure 2 - see PDF). This is similar to the data shown by Rizwan [16]. The prevalence of the type of Angle's malocclusion not being dependent on age was also stated by studies conducted by Louis et al. [17] on the population of Uganda and Tod et al. [18] on the Australian adult population. Statistically non-significant associations were also made between the Type of premolar extractions with age and gender of patients in this study (Figure 3,4 - see PDF) stating that the decisions about the type of therapeutic premolar extractions to be done are also independent of the age and gender of patients. However no study was found to support or oppose this finding. The present study found a significant association between the Type of malocclusion and the type of premolar extractions (Figure 6 - see PDF), but no study was found that could support or oppose this statement except that Vig KW et al [19] stated that the number of teeth extracted and the severity of malocclusion could influence the treatment time [20]. Limitations of the study include a restricted population group due to it being a single centred study. Future scope of the study could be improved by conducting it over a larger scale as a multi centered study.

## Conclusion

Data shows that 34.4% of subjects underwent premolar extractions amongst a total of 987 subjects who underwent orthodontic treatment. 89.4% of patients were Angle's Class I malocclusion patients, and the rest were Class II patients. However, no premolar extractions were done in Class III patients. Data also shows that 67.1% of subjects underwent all 4 first premolar extractions and 13.2% underwent only upper first premolar extractions. Thus, a significant association was found between Type of Malocclusion and the Type of premolar extractions with p < 0.05. Moreover, only 34.4% of patients underwent premolar extractions and the majority of them underwent all 4 first premolar extractions.

## Author's contribution:

R.J contributed to study conception and design, manuscript correction. A.M. contributed to data collection, analysis and interpretation of data and drafted the work. Both the authors critically reviewed the manuscript and approved the final version.

## Clinical significance:

The clinical significance of this study is to report on the prevailing trend towards extraction of teeth as a means of gaining space for orthodontic treatment.
